# Thoracolumbar Fractures: Comparing the Effect of Minimally Invasive Versus Open Schanz Screw Techniques on Sagittal Alignment

**DOI:** 10.7759/cureus.63187

**Published:** 2024-06-26

**Authors:** Elie Najjar, Mostafa Meshneb, Anish Isapure, Spyridon Komaitis, Mohamed A Hassanin, Rishi Rampersad, Belal Elnady, Khalid M Salem, Nasir A Quraishi

**Affiliations:** 1 Spinal Unit, The Centre for Spinal Studies and Surgery (CSSS) Queen's Medical Centre, Nottingham University Hospitals NHS Trust, Nottingham, GBR; 2 Department of Orthopedics and Trauma Surgery, Assiut University Hospitals, Assiut, EGY

**Keywords:** spinal fractures, open fixation, mis fixation, thoracolumbar fractures, sagittal alignment

## Abstract

Study design: This is a retrospective comparative cohort study.

Purpose: This study aims to compare the effects of minimally invasive surgery (MIS) and open surgery (OS) on global sagittal alignment (GSA) in surgically managed thoracolumbar fractures.

Overview of literature: The optimal treatment of traumatic thoracolumbar fractures (TLF) remains controversial. Both MIS techniques with polyaxial screws and OS techniques with Schanz screws have gained widespread use. The effect of each technique on the global sagittal alignment has not been reported.

Methods: From 2014 to 2021, 22 patients with traumatic TLF underwent open posterior stabilization using an open transpedicular Schanz screw-rod construct and were compared to 15 patients who underwent minimally invasive surgery using a polyaxial percutaneous pedicle screw-rod construct. The reported radiological parameters measured on preoperative supine CT scan and immediate postop standing X-ray and on final follow-up whole spine standing X-rays included pelvic incidence (PI), pelvic tilt (PT), lumbar lordosis (LL), preoperative segmental kyphosis (Preop-K), immediate post-operative segmental kyphosis (postop-Ki), final post-operative segmental kyphosis (postop-Kf), sagittal-vertica-axis (SVA), and spino-sacral angle (SSA).

Results: The average age of the OS group was 42.5 years; 5 patients had AO type B, and 17 patients had AO type A (A3 and A4) fractures. The average follow-up was 16.8 months. The average radiological parameters were: PI = 54.9°, PI-LL = 3°, PT = 17.6°, preop-K = 16.2°, postop-Ki = 8.7°, final postop-Kf = 14.3°, SVA = 4.58 cm, and SSA = 101.8°. The average age of the MIS group was 43.4 years; 5 patients had AO type B, and 10 patients had AO type A fractures. The average follow-up was 25 months. The average radiological parameters were as follows: PI = 51°, PI-LL = 8°, PT = 18°, preop-K = 18.4°, postop-Ki = 11.6°, postop-Kf = 14.3°, SVA = 6.4 cm, SSA = 106°.

Conclusion: The fixation technique did not significantly affect the final correction of the local kyphosis and global spine alignment parameters.

## Introduction

Thoracolumbar spine fractures are common, accounting for two-thirds of all spinal injuries [1]. Fifty percent of these are unstable and can result in significant disability, deformity, and neurological deficits [2]. Thoracolumbar fractures (TLFs) are more frequent in men, and the peak incidence is observed between 20 and 40 years [3]. The classification of thoracolumbar fractures has evolved over the years as the understanding of spinal biomechanics, mechanisms of injury, and identification of vertebral stability have improved [2]. In 1994, Magerl et al. [4] analyzed 1445 cases of thoracolumbar injuries and presented a comprehensive AO classification of thoracolumbar fractures based on the mechanism of injury and morphological pattern of the fracture, compression (A), tension band (B), and displacement (C) [3,5], with type A being the most common [6]. Fractures mostly affect the thoracolumbar junction due to the transition between the stiff thoracic segment and the mobile lumbar segment, with the majority (70-80%) of the patients being neurologically intact [7]. The aim of thoracolumbar fracture fixation is to restore and maintain adequate reduction, protect the neurological elements, allow early mobilization, and protect global sagittal alignment (GSA) [3,7-9].

There remains no consensus about the optimum fixation technique between the open technique using monoaxial transpedicular screws and the minimally invasive surgery (MIS) technique using polyaxial screws. The long lever arm in the Schanz screw in the open technique helps correct post-traumatic kyphosis and offers great rigidity [10]. On the other hand, Vanek et al. mentioned that surgery using minimally invasive percutaneous fixators leads to a reduction in blood loss, postoperative pain, and a faster return to previous activities [10]. But it remains unclear whether the polyaxial MIS screws can correct or maintain posttraumatic kyphotic deformity [2].

Several studies [5,11-16] have discussed the radiological outcomes related to local or segmental kyphosis correction between both techniques, but none have focused on the effect of surgical technique on global sagittal alignment. Therefore, the aim of this study is to compare the radiological spinal sagittal parameters of MIS fixation versus open Schanz screw fracture kits in thoracolumbar fractures and how they can affect global spine alignment.

## Materials and methods

Study design

We performed a retrospective analysis of radiological parameters of global sagittal alignment in patients who sustained traumatic thoracolumbar fractures and were managed surgically at our institution between 2014 and 2021.

Inclusion and exclusion criteria

According to inclusion criteria, this study selected patients with high-energy unstable fractures involving T12 to L2 (AO types A3, A4, or type B), neurologically intact, who were expected to mobilize following treatment that underwent short segment fixation performed using either the MIS or open surgery (OS) technique (one level above and one below), followed by a standing whole spine X-ray on postoperative follow-up.

The exclusion criteria were patients with pathological fractures, low-energy vertebral compression fractures secondary to osteoporosis, polytrauma with associated injuries that would prevent post-op mobilization, long segment fixation, posterior decompression, and additional anterior procedures at the fractured level, patients with multiple vertebral fractures, and patients with AO type C vertebral fractures.

Surgical technique

**Figure 1:**
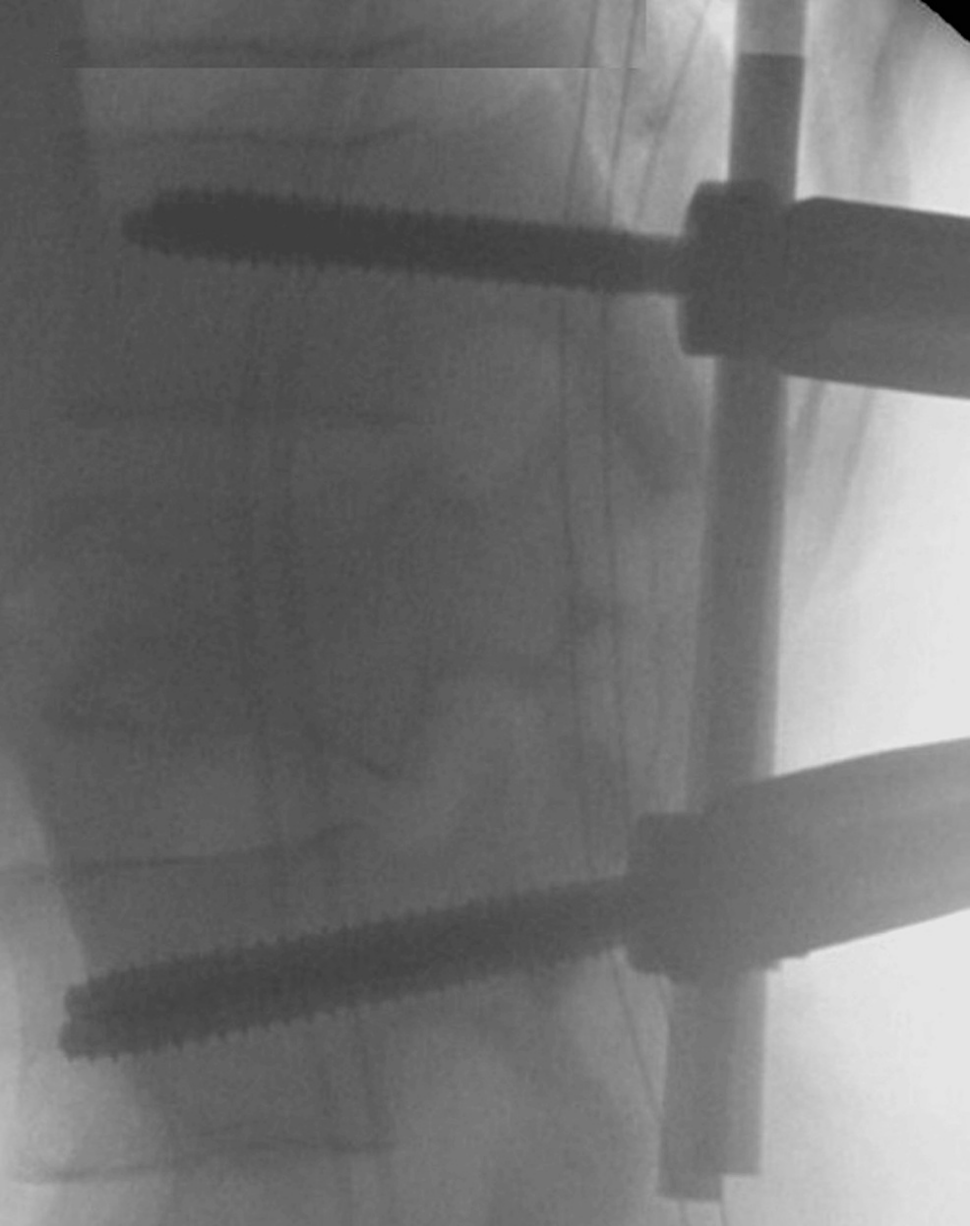
Intraoperative X-rays of minimally invasive fixation

For MIS fixation, we used polyaxial pedicle screws in all cases. Patients were positioned prone on an Allen table, which helped to restore some of the natural lordosis. Screw placement was undertaken with fluoroscopic guidance. Lordotic or neutral rods were then passed subfascially, depending on the involved level of the fractured vertebra. There were no further reduction maneuver attempts prior to securing the rods (Figure 1).

**Figure 2:**
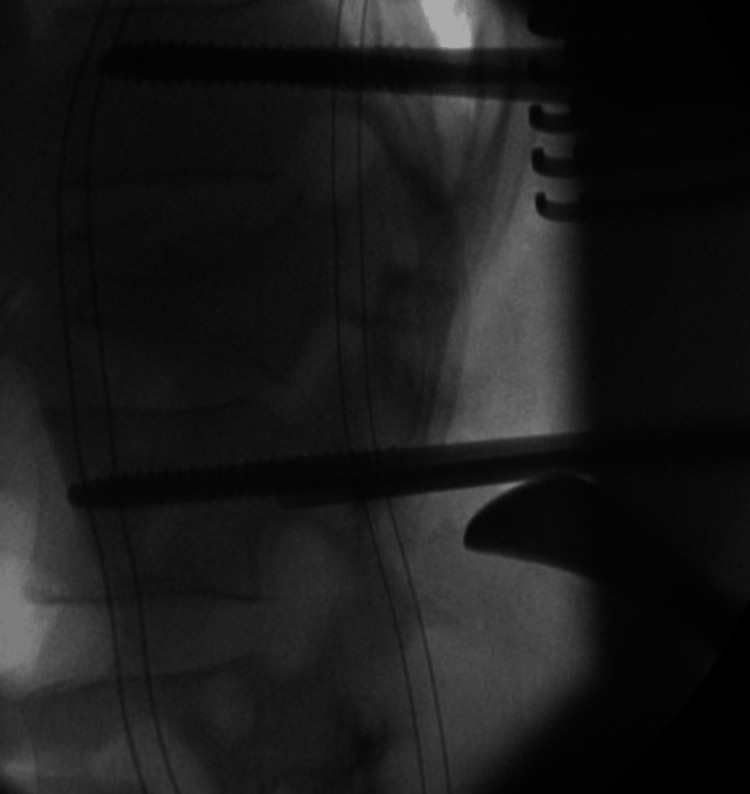
Intraoperative X-rays of open Schanz screw fixation

Patients managed by OS were also positioned prone on an Allen table to facilitate partial reduction of the kyphosis fracture segment. A midline approach was used to expose the fractured vertebra as well as the cranial and caudal levels. Instrumentation with Schanz pins of appropriate size is included on levels above and below the injury level. Appropriately sized rods were connected to the pins, followed by lordosing and distracting maneuvers. This was performed using the standard AO technique as described by Vacarro [16] (Figure 2).

Data collection

**Figure 3:**
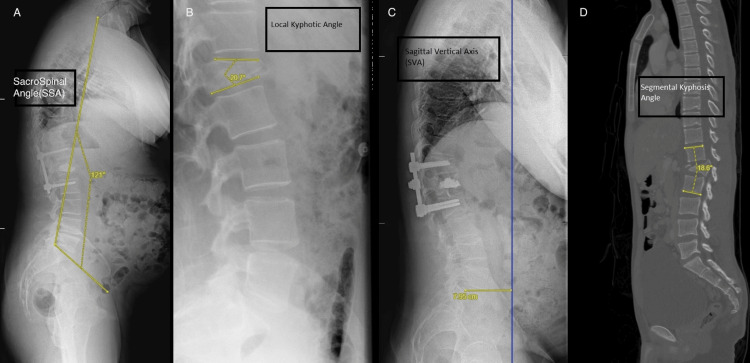
Sagittal parameter measurements (A) Postoperative Sacro-spinal angle measurement in MIS; (B) preoperative measurement of local kyphotic angle; (C) postoperative measurement of the sagittal vertical axis in open Schanz fixation; and (D) peroperative segmental kyphotic angle measurement.

The fractures were classified using the AO classification system [6]. The collected demographic data included patients' age and gender. The surgical technique was identified as either OS or MIS, in addition to the use of intercalated screws, cross-links, or cement augmentation. The final follow-up was considered at the time of the final standing whole-spine X-ray. Measured radiological parameters included preoperative segmental kyphosis (Preop-K) using a preoperative CT scan, immediate postoperative segmental kyphosis (Postop-Ki) (using standing thoracolumbar X-rays), final postoperative segmental kyphosis (Postop-Kf), pelvic incidence (PI), pelvic tilt (PT), lumbar lordosis (LL), sagittal vertical axis (SVA), and spino-sacral angle (SSA) using an upright whole spine X-ray. All measurements were done by three senior spinal surgeons using the picture archiving and communication system (PACS) software (Figure 3).

Statistical analysis

Continuous measures are summarized as means with standard deviations. Group differences were assessed using the student t-test. Nominal and categorical data were assessed using the chi-squared test. All analyses were completed using SPSS v23 (IBM Corp., Armonk, NY).

## Results

Fifteen patients were included in the MIS group (male/female ratio = 9/6) with a mean age of 43.4 years (18-66); 5 (33%) patients had AO type B fractures and 10 (66%) patients had AO type A fractures (Table 1); 7% of the cases had intercalated screws, 20% had cross-links, and 33% had vertebroplasty at the fractured level (Table 2).

**Table 1:** Summary of the patients’ demographics, injury and management characteristics MIS: minimally invasive.

Demographic data	MIS (n)	Open Schanz (n)	P-value
Cases	15	22	-
Age mean (SD)	43.4 (±18.6)	42.8 (±15.8)	0.8
Gender (M/F)	9/6	15/7	0.6
Injured level			
T 10	1	2	0.78
T 11	1	2	0.78
T 12	6	6	0.41
L 1	5	9	0.63
L2	2	3	0.97
Mechanism of injury			
Motor car accident	8	9	0.45
Fall	5	8	0.89
Sports injuries	2	5	0.47
Mean follow-up (months)	25	16.8	
AO Spine classification			
A3	4	8	0.54
A4	6	9	0.95
B1	2	2	0.68
B2	3	3	0.60

**Table 2:** Summary of the integration of intercalated screws, cross-links and vertebroplasty MIS: minimally invasive; OS: open Schanz.

Stabilization	MIS (n)	MIS (n%)	OS (n)	OS (n%)	P-value
Intercalated screw	1	7%	1	5%	0.77
Cross link	3	20%	7	31%	0.42
Vertebroplasty	5	33%	5	23%	0.47

The average follow-up was 25 months. The average radiological parameters were as follows: PI = 51°, PI-LL = 8°, PT = 18°, preop-K = 18.4°, postop-Ki = 11.6°, postop-Kf = 14.3°, SVA = 6.4 cm, and SSA = 106° (Table 3).

**Table 3:** Summary of the radiological parameters LL: lumbar lordosis; PI: pelvic incidence; PI-LL: pelvic incidence-lumbar lordosis mismatch, Postop Kf: final postoperative segmental kyphosis; Postop Ki: immediate postoperative segmental kyphosis; Preop K: preoperative segmental kyphosis; PT: pelvic tilt; SSA: spino-sacral angle; SVA: sagittal vertical axis.

Radiological parameters	MIS	OS	P-value
PI	54.9°	51°	0.3
PT	17.6°	18.3°	0.14
TK	42.5°	38.18°	0.2
LL	51.9°	42.9°	0.03
PI-LL	3°	8°	0.4
Preop K	16.2°	18.4°	0.5
Postop Ki	8.6°	11.6°	0.29
Postop Kf	14.1°	14.3°	0.95
SVA	4.5 cm	6.35 cm	0.24
SSA	101.8°	106°	0.78

A total of 22 patients underwent open Schanz screw fixation ( male/female ratio = 15/7) with an average age of 42.5 years (17-76), 5 (22%) patients had AO type B and 17 (78%) patients had AO type A (A3 and A4) fractures (Table 1); 5% of the cases had intercalated screws, 31% had cross links and 23% had vertebroplasty at the fractured level. The mean follow-up was 16.8 months. The average radiological parameters were as follows: PI = 54.9°, PI-LL = 3°, PT = 17.6°, preop-K = 16.2°, postop-Ki = 8.7°, final postop-Kf = 14.3°, SVA = 4.58 cm, SSA = 101.8° (Table 3).

Statistical analysis

There was no significant statistical difference regarding demographics, mechanism of injury, fracture level, or fracture type according to the AO classification when comparing the MIS and OS groups (Table 1). A significant improvement in post-traumatic segmental kyphosis was achieved using both techniques (MIS and OS), as demonstrated postoperatively on standing postoperative X-rays (6.5° P = 0.016 and 7.6° P = 0.003, respectively). The correction was subsequently partially lost in both cohorts by 3.9° (P = 0.15) in the MIS group and 1.8° (P = 0.55) in the OS group. There was no significant difference between the two groups with respect to the augmentation of stabilization by either intercalated screws, cross-links, or cement, as shown in Table 2. When global radiological sagittal parameters were reviewed, there was no significant difference between the two techniques, as demonstrated in Table 3.

## Discussion

To the best of our knowledge, this is the first case series comparing the effect of posterior open to minimally invasive percutaneous thoracolumbar spinal fracture fixation on global sagittal alignment. Despite showing no superiority of one technique over the other when spinal sagittal parameters are considered, thoracolumbar fractures in both groups lead to sagittal imbalances that cannot be corrected using OS or MIS techniques.

Treatment of thoracolumbar and lumbar fractures includes non-operative measures, posterior instrumented fixation or fusion, anterior column reconstruction, or combined anterior-posterior surgery. In neurologically intact patients, the decision to perform surgery is dependent on the stability and post-traumatic kyphotic deformity [17]. Posterior spinal instrumentation is the most frequent method of fixation due to its low morbidity and comorbidity [18]. However, in the absence of level I evidence studies to guide decision-making, the choice of MIS versus OS in thoracolumbar and lumbar fractures without neurological compromise is still controversial.

Both MIS and OS groups in this study had similar average age, gender, injury level, and fracture patterns according to the AO spine classification. The most common fracture levels were T12 and L1 in both groups (73% and 68%), which, according to Curfs et al. [19], increases the risk of post-traumatic kyphosis. The most common fracture type was A4 (40% in both groups). In addition, there was no significant difference in the use of intercalated screws, cross-links, or vertebroplasty for the fractured vertebra in both cohorts, which allows for a better correlation of radiologic outcomes.

While both the MIS and OS groups showed a significant reduction in segmental kyphosis in the immediate postoperative X-rays (6.5° and 7.6°), the final follow-up showed segmental collapse to a non-significant difference (3.9° and 1.8°) when compared to preoperative angles. The results were concordant with Jang et al. [20], who showed no significant differences between the two approaches regarding the restoration of vertebral body height and local kyphosis angle, concluding that both MIS and OS techniques resulted in equivalent biomechanics and clinical outcomes. In addition, Lee et al. also reported a loss of 3.1° in the MIS group and 3.5° in the OS group for segmental kyphosis [5].

To better assess the spine biomechanics, it is essential to study the global sagittal alignment, which highlights that the harmony between spinopelvic parameters maintaining the axis of gravity at a neutral location with minimal energy expenditure is indeed a state of equilibrium maintained with minimal energy expenditure [21,22]. Several studies have shown that adequate restoration of sagittal plane alignment is necessary to significantly improve the clinical outcome and avoid subsequent pseudarthrosis after spinal instrumentation [23-26]. Global sagittal balance was assessed using four parameters in our study: SVA, SSA, PI-LL, and PT.

Jackson described the sagittal vertical axis, or SVA, which corresponds to the horizontal distance between the C7 plumb line and the posterior-superior S1 corner [26]. Schwab included SVA in his sagittal modifiers, identifying 4 cm as the cut-point value [27]. The average SVA was higher in both the MIS group (4.5 cm) and the OS group (6.35 cm). SVA has its limitations as it can be influenced by the patient’s position and lower limb compensation; therefore, it does not accurately reflect the structural spinal deformity or the severity of the patient’s symptoms [28]. Alternatively, Roussouly and Nnadi [29] proposed the concept of SSA, which is an intrinsic parameter of balance. It is a parameter of overall balance because it integrates the C7 position with a pelvic parameter: the sacral slope. The SSA is a fixed angle that does not vary with compensatory changes, thus avoiding the limitations of the SVA. The mean value of the SSA in the normal population is 135° ± 8° [30]. In both the MIS and OS groups, the average SSA was lower, 101.8° and 106°, respectively, indicating a fixed sagittal imbalance.

Schwab was the first to assess the relationship between pelvic and spinal parameters based on an observational study and proposed the formula PI = LL ± 9. Recently, Le Huec and Hasegawa [30], using EOS imaging technology on 268 patients, proposed a new formula for expected LL: LL (L1−S1) = 0.54 × PI + 27.6°. Extending this analysis to our study, we find that using Schawb’s formula, both MIS (PI-LL = 3°) and OS (PI-LL = 8°) are within the normal range, while using Le Huec’s formula, both groups have a PI and LL mismatch of 5.3° and 12.25°, respectively.

PT denotes the spatial orientation of the pelvis [30]. A formula is given based on a 3D analysis of the full spine in a standing position, giving a theoretical PT = 0.44 (PI) − 11. The greater the angle of pelvic tilt, the further the center of gravity is projected behind the femoral heads, so an increased PT denotes a compensatory pelvic retroversion in response to sagittal imbalance. PT was elevated in both study groups, as the difference between the actual PT and theoretical PT was 4.4 and 6.8, respectively, indicating a compensatory mechanism.

It is worth noting that SVA, PT, and PI-LL are affected by posture and compensatory mechanisms adopted through cervical spine hyperlordosis, a decrease in thoracic kyphosis, lumbar retrolisthesis and hyperextension, pelvic retroversion, hip extension, knee flexion, and ankle extension. There was no significant difference in the radiological parameters of the MIS and OS groups in the final follow-up, and both showed signs of sagittal imbalance.

There are several limitations to this study. This is a retrospective case series with a small number of patients. The average follow-up is shorter in the OS group compared to the MIS group (25 vs. 16.8 months). The lack of a correlation between clinical outcomes and radiographic outcomes may also be a limitation. Nevertheless, this is the first study to compare the effects of MIS and OS techniques in traumatic thoracolumbar fractures on global sagittal alignment. We would recommend a randomized control study including a larger number of patients and correlating clinical and radiological outcomes.

## Conclusions

The current study's results showed that despite sagittal imbalance at final follow-up in patients with traumatic thoracolumbar fractures treated with either MIS or OS, there was no statistically significant advantage of one technique over the other in regard to correcting local kyphosis and global spine alignment parameters.
